# Systematic Review of Visual Motor Integration in Children with Developmental Disabilities

**DOI:** 10.1155/2021/1801196

**Published:** 2021-07-22

**Authors:** Blair Carsone, Katherine Green, William Torrence, Bridgett Henry

**Affiliations:** ^1^Gannon University, USA; ^2^Concordia University, USA

## Abstract

Original research articles regarding visual motor integration skills in children with developmental disabilities and the impact of occupational therapy were identified, appraised, and synthesized. Twenty-four articles were chosen for this review. Themes were noted during the critique of articles. Three themes emerged: “age,” “gender,” and “diagnosis.” Regarding the impact on visual motor integration, there was strong evidence for age, moderate evidence for gender, and strong evidence for diagnosis. Future research investigating visual motor integration in children should control for age and diagnosis.

## 1. Introduction

Visual motor integration (VMI) skills are critical for participation in meaningful activities [1]. VMI skills are necessary to complete early education activities that later influence success in kindergarten and primary school [2, 3]. For example, fundamental childhood activities such as handwriting, keyboarding, and throwing and catching a ball all utilize VMI skills. These required VMI skills surpass an isolated motor response and are complex due to the visual motor and fine motor coordination components [4]. Deficiencies in VMI skills are multifaceted, negatively impacting participation and self-concept [5, 6].

VMI is the ability to perceive visual input, process the information, and coordinate a motor response [1]. VMI skills encompass eye-hand coordination, praxis, visual perceptual skills, gross motor coordination, and fine motor coordination [7]. The ability to control hand movements through vision is necessary for a multitude of academic and nonacademic endeavors.

Children with autism spectrum disorder (ASD), brachial plexus injury (BPI), and cerebral palsy (CP) who have motor coordination and praxis difficulties are at an increased risk for VMI deficits [8]. The research has noted praxis deficits in children with ASD, BPI, and CP [9]. Six recent studies including a meta-analysis found motor coordination to be negatively impacted by ASD [10–15]. Four recent studies found motor coordination to be negatively impacted by BPI ([16–20]). Five recent studies found motor coordination to be negatively impacted by CP [21–26]. Overall, children with ASD, BPI, and CP are at an increased risk for having VMI deficits due to dyspraxia and motor coordination limitations.

Previous studies suggest that occupational therapy intervention improves VMI skills in children with disabilities as captured by the Beery VMI [4, 27–32]. Six studies found occupational therapy interventions positively impact visual motor performance in children with developmental delays [4, 27–31]. Additionally, a recent systematic review concluded that occupational therapy intervention promotes motor development, a component of VMI skills, in children from zero to five years of age [32]. These seven landmark articles confirmed consensus that occupational therapy intervention improves VMI skills in children [4, 27–32].

Occupational therapy practitioners utilize the Beery-Buktenica Developmental Test of Visual Motor Integration (Beery VMI) to assess VMI in children. VMI skills develop sequentially, and the Beery VMI adheres to this developmental sequence [33, 34]. The Beery VMI is a pen and paper assessment that requires the patient to imitate and later copy forms of increasing difficulty [35]. The first several forms are imitated, where the occupational therapist physically demonstrates the form then the child attempts to imitate it. The remaining forms are copied, where the child looks at the form and attempts to copy it [34]. The child's ability to imitate and copy the forms in a sequential order indicates the child's VMI age equivalence. The Beery VMI has been found to be psychometrically sound and is considered the standard assessment for VMI skills [34–36].

The profession of occupational therapy strives to provide evidence-based practice. The utilization of standardized measures such as the Beery VMI supports best practice as it allows the effectiveness of occupational therapy intervention to be assessed over time [37]. The effectiveness of occupational therapy practice through standardized measures is essential for occupational therapy professionals to continue to make informed, evidence-based decisions about best practice and the effectiveness of intervention techniques.

The contemporary research regarding VMI in children with developmental disabilities and the impact of occupational therapy is limited in both quantity and quality (i.e., generalizability). Additionally, there is no reference available denoting the expected changes in Beery VMI scores following occupational therapy intervention. Due to the lack of current evidence on the topic, this review of literature broadly evaluated the current understanding of VMI scores of children with developmental disabilities. This literature review addressed the gap by evaluating the contemporary research and answers the question, “What factors influence VMI scores of children with developmental disabilities?”.

## 2. Review of the Literature

This literature review explored the factors that influence VMI of children with developmental disabilities. Relevant articles were identified by searching EBSCO, Google Scholar, and Sage Journals through Concordia University's Research Databases based on the availability of search engines. Search terms included various combinations of the following: “visual motor integration,” “children,” and “developmental disabilities”.

Criteria for inclusion in this review were the following: (a) articles were original scientific reports of studies, (b) articles were focused on visual motor integration in children including those two to five years of age, (c) articles were published in English-language peer-reviewed journals, (d) articles were available in full-text, and (e) articles were published during or after 2010. Criteria for exclusion in this review were the following: (a) articles that were dissertations or theses, (b) articles that did not include visual motor integration or children, (c) articles that were published in non-English language, (d) published abstracts, (e) articles published before 2010, and (f) articles that focused on children with intellectual disabilities (ID). Much of the research on developmental disabilities and VMI has focused on children with ID. The authors determined that the research and understanding for this specific diagnosis was adequate due to published meta-analyses on the subject [38]. Therefore, ID were not included in this literature review.

The literature search produced 986 titles which were first scanned for relevance. Two hundred thirty-five relevant abstracts were evaluated with 54 being selected and reviewed. After applying inclusion and exclusion criteria, 24 articles were chosen for this review.

Themes were noted during the critique of articles. The themes were not derived through a specific qualitative methodological approach; rather they were generated based on recurring similarities. As such, articles were categorized into three demographic themes: “age,” “gender,” and “diagnosis”.

### 2.1. Age

Throughout the first five years of life, children achieve gross and fine motor milestones that enable them to interact with the world around them. Each milestone provides the foundation for the achievement of successive milestones as the child progresses towards autonomy [39]. When milestones are delayed or not achieved, the child is considered to have an early marker for further impaired development [40]. VMI developmental milestones begin at three months of age, when an infant can track an object in supine [41]. Milestones progress as the child develops with the Beery VMI accurately assessing VMI skills starting at two years of age [42].

Six recent studies explored the implications of age and VMI performance in children. Two studies included children five years and older [43, 44]. Three studies investigated performance of children starting at four years of age [45–47]. One study included children as young as three years six months [48]. All studies were at a consensus that age impacted VMI scores [43–48].  Table 1 provides a summary of the findings.

Recently, one study utilized the Beery VMI to examine the relationship between motor coordination, visual perception, and executive function to VMI skills of four to six-year-old preschoolers [46]. VMI skills of children increased at four years of age and peaked at five years of age [46]. These findings, however, may not be applicable for all populations. Research investigating children between the ages of four and 12 years old with Williams Syndrome and those of typical development concluded that age impacted VMI scores of children who were typically developing, but not children with Williams syndrome [47]. Overall, these studies illustrate that age may have varying influences on different populations of children.

Two studies investigating preschool children aged three years six months to five years, respectively, concluded that age and geographic location affected VMI scores [44, 48]. Similar findings were noted previously when 60 to 72-month-old children were studied [43]. Additionally, this study concluded that age and socioeconomic status, commonly associated with location, were found to impact VMI scores. Overall, these three studies illustrate how age as well as location/socioeconomic status impact the VMI scores of children.

Lastly, one recent study explored VMI scores of children four years to seven years of age in relation to intervention [45]. Age was found to impact VMI score, while occupational therapy intervention was concluded to still be the superior VMI intervention when compared to the latest iPad intervention [45]. Overall, the six studies were at a consensus that age impacts VMI scores and needs to be accounted for during research and clinical practice [43–48].

### 2.2. Gender

It is theorized that gender-related differences in motor learning and performance impact VMI skills [49, 50]. Older studies have shown fine motor and visual motor skills to be similar between sexes or better in females [51]. The inconsistencies in the aforementioned body of literature do not allow for conclusive statements regarding VMI skills in preschool children.

The current literature clarifies previous findings. Four of the five studies indicate significant differences between gender while one study found no significant difference [52–56].  Table 2 provides a summary of the findings.

Four of the five studies noted differences between genders [52–54, 56]. Specifically, one study reported that females had greater VMI abilities than males [52]. While another study concluded that males were at a higher risk for VMI deficits [54]. Another study noted gender differences favoring females before a stimulating activity intervention; however, males superseded female skills after the intervention [56]. Therefore, although gender differences have been reported, there are inconsistencies regarding which gender has superior VMI skills [52, 54, 56].

Conversely, one study found no significant differences between genders for children four to 14 years old with severe language impairment [55]. There was no significant difference in mean performance between males and females; however, children with poor VMI skills also had poor receptive language skills [55]. VMI deficits in children with a language impairment component are common due to the interconnection between motor, visual, and integration difficulties. Overall, gender may impact VMI skills in children, but no definitive conclusions can be drawn at this time [53, 55, 56].

### 2.3. Diagnosis

In the United States, it is reported by the Centers for Disease Control and Prevention [57] that one in six children has one or more developmental disabilities or other developmental delays. Examples of developmental disabilities include, but are not limited to, ASD, BPI, and CP. Additional diagnoses that are less common can also impact VMI skills in children.

Findings in the literature and the author's clinical experiences highlight the common association of ASD, BPI, and CP with VMI deficits. Children with ASD, BPI, and CP who have low motor abilities are at an increased risk for VMI deficits [8]. The neurological and physical aspects of the diagnoses contribute to the impaired fine motor and visual motor components necessary for VMI skills. The current literature illustrates the VMI deficits experienced by children with ASD, BPI, and CP [16, 58–64].

### 2.4. ASD

Three recent studies that focused on children with ASD reiterated the implications of the diagnosis on VMI skills [61–63]. Two studies specifically addressed VMI while one study explored the kinematic aspect of VMI skills of children with ASD [61–63]. Overall, the studies were at a consensus that ASD negatively impacts VMI skills [61–63].  Table 3 provides a summary of the findings.

Most recently, a study examined Beery VMI performance in males with ASD compared to males with typical development between the ages of three and 23 years old [63]. The study found a considerable number of the males with ASD experienced difficulties in performing VMI-related tasks compared to the males who were typically developing [63]. Similarly, previous research investigated the implications of ASD and attention deficit hyperactivity disorder (ADHD) on working memory and VMI when compared to children who are typically developing aged four to 18 years old [62]. It was concluded that both children with ASD and ADHD demonstrated working memory and VMI deficits [62]. Overall, both studies illustrate deficient VMI skills in children with ASD.

One study explored kinematics of children three to seven years old with ASD [61]. Children with ASD demonstrated increased variability in the preparation phase of movement due to VMI deficits that allow the integration of visual information to inform movement planning and execution [61]. Overall, the studies were at a consensus that ASD negatively impacts VMI scores [61–63].

### 2.5. BPI

Only two recent studies explored the VMI skills of children with BPI. The two studies that focused on children with BPI alluded to the possible implications of the diagnosis on VMI skills [16, 64]. Overall, the studies were at a consensus that BPI may have a negative impact on VMI skills [16, 64].  Table 4 provides a summary of the findings.

Most recently, a study investigated quality of life in children with BPI under three years of age based on parent perception [64]. It was concluded that perception was impacted by the side affected (i.e., right or left) and severity of BPI [64]. Severity of BPI is linked to negative impacts on physical functioning including VMI tasks [65]. Similarly, a study explored coordination and balance deficits in children with BPI between the ages of five to 15 years old [16]. More than half the participants indicated disability for bilateral coordination or body coordination on a self-report measure of physical disability [16]. Overall, these studies demonstrated the functional limitations experienced by children with BPI, which can impact VMI skills [16, 64].

### 2.6. CP

Three recent studies included in this review that focused on the VMI of children with CP reiterated the implications of the diagnosis on VMI skills and the improvements in VMI skills with intervention. Two of the three studies investigated augmented biofeedback and traditional therapy as a specific intervention to address VMI skills in children with CP [58, 59]. The third study focused on the functional implications of the condition [60]. Overall, the studies included in this review were at a consensus that CP negatively impacts VMI skills [58–60].  Table 5 provides a summary of the findings.

Most recently, two studies investigated eye-hand coordination in children with CP between the ages of five to eight years old before and after receiving varied interventions [58, 59]. Augmented biofeedback with traditional therapy intervention significantly improved VMI when compared to children that only received one intervention [58, 59]. Overall, the studies demonstrated the impact of CP and intervention on VMI scores.

The third research study explored visual perceptual intervention on VMI skills and activities of daily living performance of children with CP [60]. Visual perceptual intervention had a positive influence on the VMI skills and activities of daily living performance of children with CP [60]. Overall, the studies illustrate that deficient VMI skills in children with CP can be improved through intervention [58–60].

### 2.7. Other Diagnoses

Five recent studies explored the implications of other diagnoses (e.g., coordination disorders, prematurity, and HIV) and VMI performance in children [66–70]. Three studies examined VMI deficits in children with coordination disorders [66, 68, 70]. One study reported VMI deficits in children of very preterm and very low birth weight [67]. Another study reported a negative impact on VMI skills in children with primary stereotypic movement disorder [70]. Overall, the literature was at a consensus that diagnosis impacts VMI skills [66–70].  Table 6 provides a summary of the findings.

Three studies examined VMI deficits in children with coordination disorders [66, 68, 70]. Participation in activities was positively correlated with motor ability as captured by the VMI score in children with DCD [68]. However, no difference was found between children with primary stereotypic movement disorder and children of typical development in VMI total score [70]. Similarly, no correlation between motor coordination and IQ was found in children with 22q11-deletion syndrome, but a significant correlation was found between IQ scores and Beery VMI scores [66]. Overall, VMI skills varied depending on the diagnosis studied.

Additionally, two studies investigated various subpopulations of children and found VMI deficits in each population [67, 70]. Children of very preterm and very low birth weight demonstrated impaired motor functioning and a range of deficits in attention and VMI skills [67]. For children infected with HIV, a moderate relationship was found between VMI scores, school attendance, and mother's level of education [69]. Overall, these studies illustrate how children with developmental disabilities often have VMI deficits specific to their diagnosis.

## 3. Discussion

The systematic review investigated the VMI of children with developmental disabilities. The literature was at a consensus that age effects VMI [43–48]. The literature reported conflicting information regarding the impact of gender on VMI [52–56]. The literature was at a consensus that diagnosis impacts VMI [66–70]. Specifically, ASD, BPI, and CP are associated with VMI deficits [16, 58–64].

These findings are clinically relevant and follow the three major themes of this literature review: age, gender, and diagnosis. First, the findings reiterate the importance of age-based norms when assessing VMI skills. Standardized assessments, such as the Beery VMI, that utilize age-based norms and describe performance with age equivalence are recommended. Occupational therapy practitioners can then use this quantitative data to create measurable goals. Therapeutic activities can also be selected that best promote the acquisition of VMI milestones through the just-right challenge. Second, any VMI evaluation tool needs to be assessed to assure that there is no gender bias (i.e., the Beery VMI). Third, diagnosis can impact VMI and should therefore be assessed by pediatric rehabilitation professionals. VMI can be easily overlooked in pediatric practice when children have multiple deficits; however, VMI can impact multiple areas of occupation. The improvement of VMI can lead to positive changes in occupational performance and should be included during evaluation. Overall, these findings impact current clinical practice.

These findings are also relevant to future research. First, future research will need to control for age and effects of maturation. This can be accomplished by establishing age groups and limiting the length of intervention (e.g., six months). Diagnosis can be addressed by researching specific populations or controlling for diagnosis as a covariate. At this time, it is not necessary to control for gender as long as an appropriate assessment is utilized (e.g., the Beery VMI). With these recommendations in mind, future research should investigate the effects of intervention on the VMI of children with developmental disabilities.

## 4. Conclusion

This literature review broadly explored the VMI of preschool-aged children with developmental disabilities from which three main themes emerged: age, gender, and diagnosis. The literature was at a consensus that age effects VMI [43–48]. The literature reported conflicting information regarding the impact of gender on VMI [52–56]. The literature was at a consensus that diagnosis impacts VMI [66–70]. Specifically, ASD, BPI, and CP are associated with VMI deficits [16, 58–64].

At this time, it is recommended that both clinicians and researchers take note of the impact of age and diagnosis on VMI. Future studies may clarify the impact of gender on VMI, but the use of an unbiased assessment (e.g., the Beery VMI) helps to control for this potentially confounding variable. Future research should focus on interventions for VMI to improve the efficacy of services provided to children with developmental disabilities. The literature included in this review was mostly descriptive in nature and did not investigate the efficacy of occupational therapy interventions to promote best practice.

## Figures and Tables

**Table 1:** Summary of age and visual motor integration performance studies.

Authors	Descriptions	Subjects	Findings
Coutinho et al. [45]	Explored the effectiveness of iPad applications in children four years to seven years old with poor VMI skills	20 children between the ages of four years and seven years 11 months with poor VMI skills	No significant difference was found
Ercan et al. [43]	Utilized the Beery VMI to investigate the VMI skills of children of varying socioeconomic statuses	Children between the ages of 60 months to 72 months from low and high economic status	Children 67 to 72 months and those of high socioeconomic status had better VMI scores
Fang et al. [46]	Utilized the Beery VMI to examine the relationship between motor coordination, visual perception, and executive function to VMI skills	151 children between the ages of four and six years of age	VMI skills of children increased at four years of age and peaked at five years of age
Heiz and Barisnikov [47]	Utilized the Beery VMI to assess the VMI differences between children with Williams syndrome and those of typical development	26 participants with Williams syndrome aged six to 41 years old and 154 children of typical development between the ages of four and 12 years old	Age did not influence ability in the children with Williams syndrome, whereas age was a significant factor for those of typical development
Ng et al. [48]	Utilized the Beery VMI to examine performance differences in preschool children	288 children between three years six months to five years 11 months from 54 preschools	Hong Kong cohort scored significantly better when compared to the normative sample of preschoolers in the United States
Visser et al. [44]	Utilized the Beery VMI to investigate skills of five-year-old English-speaking children who reside in South Africa	68 children between the ages of five years six months to five years 11 months from seven schools	VMI skills of children scored below average in all the subtests, but compared well to the normative sample established in the United States

*Note.* The abbreviations “Beery VMI” and “VMI” represent the “Beery-Buktenica Developmental Test of Visual Motor Integration” and “visual motor integration”, respectively.

**Table 2:** Summary of gender and visual motor integration performance studies.

Authors	Descriptions	Subjects	Findings
Coallier et al. [52]	Utilized the Beery VMI to assess children in Canada and compare to the norms established United States sample	151 children ages of five to six years old from kindergarten classes in Quebec, Canada	Significant gender difference was found where females obtained higher mean scores than males
Cui et al. [53]	Utilized the Beery VMI to determine VMI performance in Chinese children compared the normative sample established in the United States	365 children ages of three to 12 years old from six public schools in Shanghai, China and Ningbo, China	Gender and residence differences were significantly greater for the Chinese children that were female and from Shanghai
Memisevic and Hadzic [54]	Utilized the Beery VMI to assess the relationship between VMI and articulation disorders in children	286 children ages of three to six years old from public schools in Canton Sarajevo	Significant gender difference was found for both VMI and articulation disorders where males are at a higher risk compared to females
Nicola and Watter [55]	Utilized the Beery VMI to investigate the VMI skills of children four to 14 years old with severe language impairment	100 children ages of four to 14 years old with language scores at least two standard deviations below the mean in Australia	No significant difference in mean performance between males and females; however, children with poor VMI skills also had poor receptive language skills
Singh et al. [56]	Utilized the Beery VMI to investigate the impact of stimulating activities on Haryana children between the ages of two and three years old	100 children between the ages of two and three years old were randomly selected from two Indian villages of Hisar District of Haryana, Gangva as the control and Muklan the experimental group	Initially, there were gender differences favoring females before the stimulating activities intervention; however, males superseded female skills after the intervention

*Note.* The abbreviations “Beery VMI” and “VMI” represent the “Beery-Buktenica Developmental Test of Visual Motor Integration” and “visual motor integration”, respectively.

**Table 3:** Summary of autism spectrum disorder and visual motor integration performance studies.

Authors	Descriptions	Subjects	Findings
Dowd et al. [61]	Explored kinematics of children three to seven years old with ASD	13 children with ASD between the ages of three and seven years old were matched to a group of 13 children of typical development in Australia	Children with ASD demonstrated increased variability in the preparation phase of movement due to VMI deficits
Englund et al. [62]	Investigated the implications of ASD and ADHD on working memory and VMI when compared to typically developing children	49 children with ADHD, 33 children with ASD, and 79 children of typical development aged four to 18 years old were compared in the United States	Both children with ASD and ADHD demonstrated working memory and VMI deficits
Green et al. [63]	Examined Beery VMI performance in males with ASD compared to males with typical development between the ages of three to 23 years old	56 participants with ASD and 36 participants of typical development were compared with multiple versions of the Beery VMI being administered throughout the longitudinal study conducted in the United States	No significant group differences were observed between the three versions for either group; however, a considerable number of males with ASD experienced difficulties compared to typically developing males in performing VMI-related tasks

*Note.* The abbreviations “ASD,” “ADHD,” “Beery VMI,” and “VMI” represent “autism spectrum disorder,” “attention deficit hyperactivity disorder,” “Beery-Buktenica Developmental Test of Visual Motor Integration,” and “visual motor integration,” respectively.

**Table 4:** Summary of brachial plexus injury and visual motor integration performance studies.

Authors	Descriptions	Subjects	Findings
Bellows et al. [16]	Explored coordination and balance deficits in children with BPI between the ages of five and 15 years old	39 children with birth-related BPI between the ages of five and 15 years old in British Columbia	More than half the participants indicated disability for bilateral coordination or body coordination on a self-report measure of physical disability
van der Holst et al. [64]	Investigated quality of life and motor function in children with BPI under three years of age based on parent perception	59 children with a median age of 18 months participated from the Netherlands	Perception was impacted by side, severity of BPI, and motor function of the upper extremity

*Note.* The abbreviation “BPI” represents “brachial plexus injury,” respectively.

**Table 5:** Summary of cerebral palsy and visual motor integration performance studies.

Authors	Descriptions	Subjects	Findings
Alwhaibi et al. [58]	Investigated eye-hand coordination in children with CP between the ages of five and eight years old before and after receiving varied interventions	45 children between the ages of five to eight years old with spastic hemiplegia were randomized into three groups	Augmented biofeedback with traditional physical therapy intervention significantly improved VMI when compared to children that only received one intervention
Alwhaibi et al. [59]	Examined VMI in children with CP between the ages of five and eight years old before and after receiving varied interventions	45 children between the ages of five and eight years old with spastic hemiplegia were randomized into three groups	Augmented biofeedback with traditional physical therapy intervention significantly improved eye hand coordination when compared to children that only received one intervention
Cho et al. [60]	Explored visual perceptual intervention on VMI and activities of daily living performance of children with CP	56 children with CP between four and seven years old	Visual perceptual intervention had a positive influence on the VMI and activities of daily living performance of children with cerebral palsy

*Note.* The abbreviations “CP” and “VMI” represent “cerebral palsy” and “visual motor integration,” respectively.

**Table 6:** Summary of other diagnoses and visual motor integration performance studies.

Authors	Descriptions	Subjects	Findings
Duijff et al. [66]	Investigated intelligence and VMI in children with 2q11-deletion syndrome the ages of five years zero months to five years 11 months old through administration of the Beery VMI	65 children between the ages of the ages of five years zero months and five years 11 months old with 22q11-deletion syndrome	A significant correlation was found between IQ scores and Beery VMI scores; however, no correlation between motor coordination and IQ was found
Geldof et al. [67]	Investigated VMI in children of very preterm and very low birth weight through administration of the Beery VMI	106 children at five years six months old who were born before 32 weeks gestational age and weighed less than 1500 grams	Approximately 1/3 of the children demonstrated impaired motor functioning and a range of deficits in attention and VMI skills
Jarus et al. [68]	Investigated participation patterns of children with and without DCD	25 children with DCD between the ages of the ages of five to seven years old and 25 children without DCD between the ages of the ages of five years and seven years old	Preference to participate in activities was positively correlated with motor ability as captured by the VMI score
Odejayi et al. [69]	Investigated VMI delays in children infected with HIV	71 children between the ages of five years zero months and five years 11 months old who were HIV positive through vertical transmission	A moderate relationship was found between VMI scores, school attendance, and mother's level of education
Valente et al. [70]	Investigated the motor profiles of children with primary stereotypic movement disorder and compare to children of typical development	26 children between 35 months and 76 months old with primary stereotypic movement disorder were compared to 27 children of typical development between 36 and 59 months old	No difference was found between children with primary stereotypic movement disorder and children of typical development in VMI total score

*Note.* The abbreviations “VMI,” “Beery VMI,” and “DCD” represent “visual motor integration,” “Beery-Buktenica Developmental Test of Visual Motor Integration,” and “developmental coordination disorder,” respectively.

## Data Availability

Carsone, B. (2021). Occupational therapy intervention and Beery VMI scores of children with autism spectrum disorder, brachial plexus injury, and cerebral palsy (Publication No. 28318846) [Doctoral dissertation, Concordia University]. ProQuest Dissertations & Theses Global.
